# Context-Dependent Tumorigenic Effect of Testis-Specific Mitochondrial Protein *Tiny Tim 2* in Drosophila Somatic Epithelia

**DOI:** 10.3390/cells9081842

**Published:** 2020-08-06

**Authors:** Cristina Molnar, Anxela Louzao, Cayetano Gonzalez

**Affiliations:** 1 Institute for Research in Biomedicine (IRB Barcelona), The Barcelona Institute of Science and Technology, Baldiri Reixac, 10, 08028 Barcelona, Spain; cristina.molnar@irbbarcelona.org (C.M.); anxela.louzao@gmail.com (A.L.); 2 Catalan Institution for Research and Advanced Studies (ICREA), 08010 Barcelona, Spain

**Keywords:** drosophila cancer model, cancer/testis genes, hyperplasia

## Abstract

We have undertaken a study towards understanding the effect of ectopic expression of testis proteins in the soma in Drosophila. Here, we show that in the larval neuroepithelium, ectopic expression of the germline-specific component of the inner mitochondrial translocation complex *tiny tim 2* (*ttm2*) brings about cell autonomous hyperplasia and extension of G2 phase. In the wing discs, cells expressing ectopic *ttm2* upregulate Jun N-terminal kinase (JNK) signaling, present extended G2, become invasive, and elicit non-cell autonomous G2 extension and overgrowth of the wild-type neighboring tissue. Ectopic *tomboy20*, a germline-specific member of the outer mitochondrial translocation complex is also tumorigenic in wing discs. Our results demonstrate the tumorigenic potential of unscheduled expression of these two testis proteins in the soma. They also show that a unique tumorigenic event may trigger different tumor growth pathways depending on the tissular context.

## 1. Introduction

Cancer/Testis (CT) genes are a heterogeneous group of genes that under normal conditions are predominantly expressed in testis and trophoblast, but are atypically upregulated in cancers of various histological origins [1,2,3]. Because the blood-testis barrier creates an immune privileged site [4] some of the proteins encoded by CT genes are antigenic and elicit humoral and cellular immune responses when expressed in somatic tumors. Indeed, it was through the autoimmune response against such CT antigens in cancer patients that the first CT genes were identified [5,6]. The current CT database (www.cta.lncc.br) includes more than 250 CT genes belonging to about 150 gene families [5]. In addition, recent studies based on integrating data from multiple databases have identified over 800 CT genes defined as such on the basis of their expression being limited to testis and neoplastic cells, regardless of their immunogenicity [7,8].

Some CT proteins have become instrumental in oncology as candidates for immunotherapy [9,10,11,12], or as biomarkers to predict recurrence or to discern malignancy grades [13]. However, beyond these applications, the key standing question regarding CT genes is whether they are just coincidental markers of tumor progression or significant mediators of tumorigenesis. It has been proposed that CT genes could play important roles in malignant growth by contributing certain germline traits like immortality, invasiveness, hypomethylation and others [2]. Indirect evidence in this regard is tantalizing, but so far, only a handful of CT genes have been implicated in tumor growth and a strong body of evidence demonstrating a function for CT genes in human cancer is still lacking [8,14,15,16,17].

In the context of a long-term study using Drosophila towards understanding the effect of ectopic expression of testis proteins in the soma, we have found that Tiny tim 2 (Ttm2) and Tomboy20, have tumorigenic effects when expressed in somatic epithelia. Ttm2 and Tomboy20 are testis-specific components of the translocases of outer (TOM) and inner (TIM23) membrane complexes that translocate nuclearly encoded mitochondrial proteins into mitochondria [18,19,20]. Larval neuroepithelial cells expressing *ttm2* overproliferate and fail to differentiate into medulla neuroblasts (NBs). Imaginal wing disc epithelial cells expressing *ttm2* or *tomboy20* invade and induce non-autonomous massive overgrowth of the nearest wild-type epithelium. Our results show that precise control of somatic repression of these testis-specific genes is necessary to maintain tissue homeostasis and provide direct evidence substantiating the tumorigenic potential of unscheduled expression in somatic cells of two testis-specific proteins.

## 2. Materials and Methods

### 2.1. Fly Stocks

The following fly strains were used in this study: the FlyORF lines *UAS-ttm2-HA, UAS-tomboy20-HA*, and *UAS-Tom20-HA* [21], *UAS-GFP*, *UAS-p35* (Bloomington Drosophila Stock Center (BDSC) #5072), *hh-Gal4* [22], *nub-Gal4* [23], *c855a-Gal4* (BDSC #6990)*, TRE-GFP* (BDSC #59010) and the Fly-FUCCI reporters Ubi-GFP.E2f1^1−230^ and Ubi-mRFP1.NLS.CycB^1−266^ (BDSC #55123 and #55124). The wild-type strain used was *w^1118^*. All crosses, including controls, were maintained at 25 °C.

### 2.2. Immunohistochemistry

Immunostaining of whole larval brains and wing discs was performed as described in [24,25]. Briefly, wing discs and brains were dissected in phosphate-buffered saline (PBS), fixed in 4% formaldehyde, rinsed in PBS–0.3% Triton X-100 (PBST), and blocked for 60 min in PBST with 10% fetal calf serum (PBSTF). Primary and secondary antibodies were incubated in PBSTF overnight at 4 °C. Primary antibodies used in this study include rabbit anti-Dcp1 (1:100; Cell Signalling, Danvers, MA, USA), rat anti-DE-cadherin (DCAD2, 1:100, Developmental Studies Hybridoma Bank (DSHB)), mouse anti-Dac (mAbdac1-1, 1:100, DSHB), mouse anti-FasIII (7G10; 1:100, DSHB), mouse anti-Dlg (4F3; 1:100, DSHB), rabbit anti-Patj (1:500; gift from E. Knust), mouse anti-Sdt (1:500; gift from E. Knust), rabbit anti-Crb (1:1000; gift from E. Knust), rabbit anti-aPKC (1:500; Sigma, Kawasaki, Kanagawa, Japan), rabbit anti-HA (1:1000, Active Motif, Carlsbad, CA, USA) and rat anti-PH3 (1:2000, Active Motif). We used Alexa Fluor secondary antibodies (1:1000, Life Technologies, Carlsbad, CA, USA). DNA was stained with 4′,6-diamidino-2-phenylindole (DAPI). F-actin was stained with Phalloidin-Rodamine. Larval brains and wing discs were mounted in Vectashield (Vector Laboratories, Burlingame, CA, USA). Images were acquired with an SP8 Leica confocal image microscope and processed in Adobe Photoshop CS6 (Adobe, San Jose, CA, USA) and ImageJ (National Institutes of Health, Bethesda, MN, USA).

### 2.3. Quantification and Statistical Analysis

Percentage of the area covered by Dcp1 positive cells and quantification of nuclear sizes in wing discs, and quantification of neuroepithelium (NE), medulla (MED) and lamina (LAM) widths in larval brain lobes were carried out using single focal plane images acquired with an SP8 Leica confocal image microscope, and by measuring areas in the wing discs or length in the brain lobes using ImageJ software. The mitotic index was calculated as density of PH3-labelled cells in wing discs using stacks of 10 focal planes per wing discs acquired with an SP8 Leica confocal image microscope, and the images were z-projected using ImageJ software. Quantification of FasIII staining was carried out using ImageJ to calculate the mean grey values of each Region Of Interest (ROI) in Z-sections obtained from stacks of 100 focal planes per wing disc acquired with an SP8 Leica confocal image microscope.

All individual measurements were represented in scatter dot plots showing mean with SD. *p* values were calculated by nonparametric Mann–Whitney U tests using GraphPad Prism 8.00 for MacOS X (GraphPad Software, La Jolla, CA, USA) (www.graphpad.com).

## 3. Results

### 3.1. Ectopic Expression of Ttm2 Induces Hyperplasia in the Neuroepithelium

To determine the effect of ectopic expression of the testis-specific mitochondrial translocator complex proteins Ttm2 and Tomboy20 in the larval brain we used *c855-Gal4* that at larval stages drives expression in the brain, the optic lobes notably in the neuroepithelial cells of the outer optic anlage, and in different regions of the wing, eye, and leg discs [26]. *c855-Gal4>UAS-ttm2* and *c855-Gal4>UAS-tomboy20* (henceforth referred to as *c855>ttm2* and *c855>tomboy20*) individuals present developmental delay and early pupae lethality. Immunofluorescence with antibodies against the Hemagglutinin (HA) tag confirms a fairly ubiquitous expression of HA-Ttm2 and HA-Tomboy20 over the brain lobe including lamina, neuroepithelium (NE), medulla, and central brain in *c855>ttm2* and *c855>tomboy20* larvae (Supplementary Figure S1). We found no evidence of apoptosis in *c855>ttm2* and *c855>tomboy20* larval brains.

**Figure 1:**
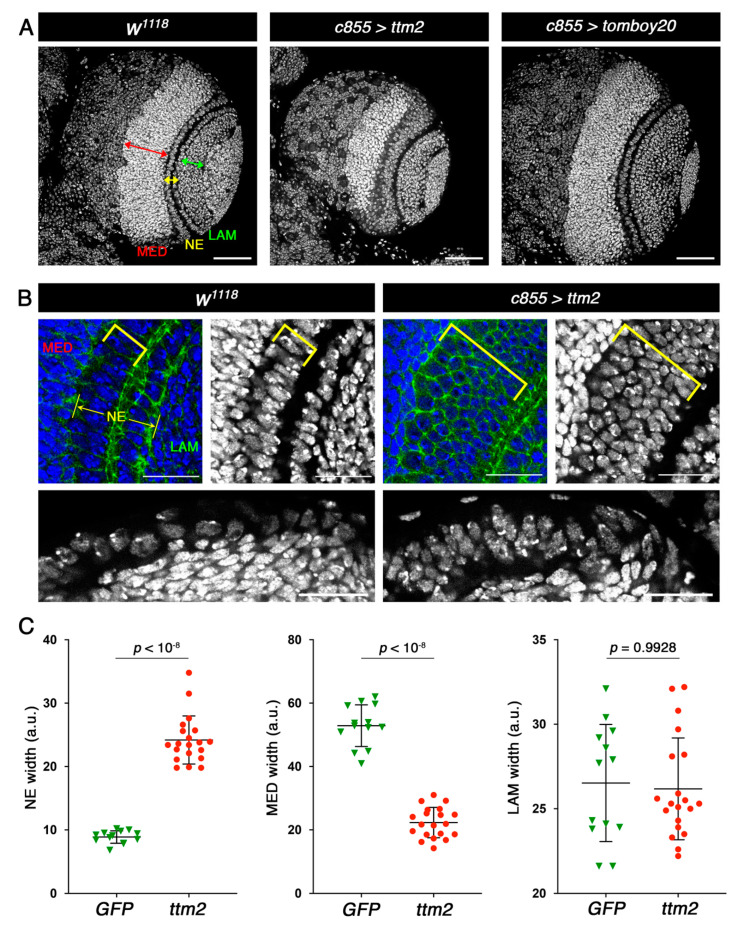
Ectopic expression of Ttm2 causes hyperplasia in the larval neuroepithelium. (**A**) Control brain lobes (*w^1118^*), and brain lobes expressing *ttm2* (*c855>ttm2*) and *tomboy20* (*c855>tomboy20*) stained with DAPI (grey). Medulla (MED), neuroepithelium (NE), and lamina (LAM) are labelled by red, yellow and green arrows, respectively. The NE is broader than control (*w^1118^*) in *c855>ttm2,* but unaffected in *c855>tomboy20* brain lobes. Scale bar, 50 µm. (**B**) High magnifications of the NE region in frontal (upper panels) and cross sections (lower panels) from *w^1118^* and *c855>ttm2* brains lobes stained with DAPI (blue and gray) and anti-DE-cadherin antibodies (green). Yellow brackets show the medulla side of the NE. Scale bar, 20 µm. (**C**) Mean, SD, and scattered plots of the width of NE, MED and LAM in control *c855>GFP* (GFP; green; *n* = 13) and *c855>ttm2* (ttm2; red; *n* = 20) brain lobes. Differences in NE and MED sizes are highly significant.

Staining with DAPI did not reveal any visible effect of ectopic *tomboy20* in larval brain development (Figure 1A). However, *ttm2* expression has a distinct effect on NE and medulla development (Figure 1A–C; yellow and red arrows, respectively). Mean NE width in *ttm2* expressing brains (*c855>ttm2*) is significantly larger than in wild-type control brains (24.19 ± 3.79 and 8.87 ± 0.97, respectively; *p* < 10^−8^) while in turn mean medulla widths are significantly smaller in *ttm2* expressing brains than in control brains (22.30 ± 4.78 and 52.88 ± 6.58, respectively; *p* < 10^−8^). No significant changes were observed in lamina width between control (27.94 ± 5.06) and *c855>ttm2* brains (26.18 ± 3.01; *p* = 0.6043) (Figure 1C).

**Figure 2:**
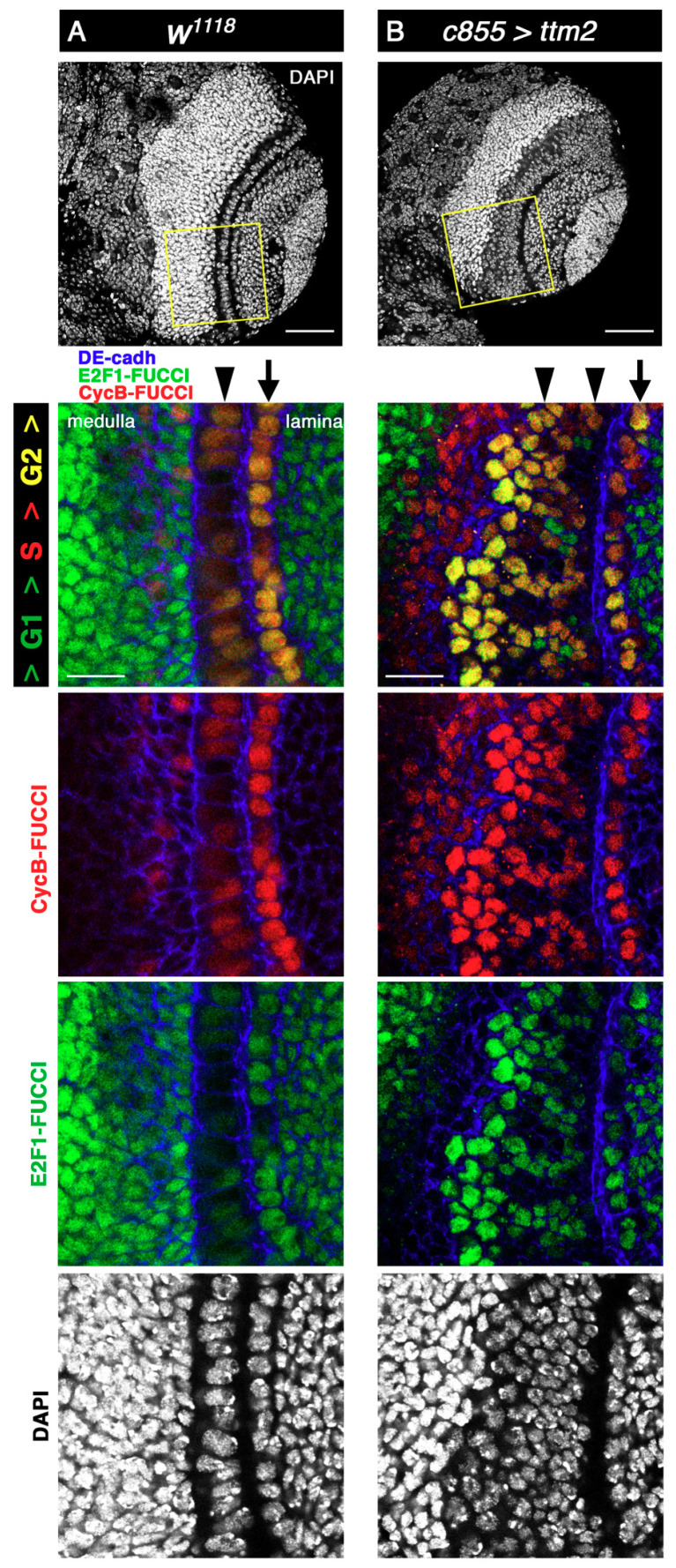
The hyperplastic Ttm2-expressing NE presents a significant extension of G2. (**A**) Control and (**B**) *ttm2*-expressing larval brain lobes. The two upper panels show low magnification views of the entire brain lobe stained with DAPI (grey). The lower panels show high magnification views of the FlyFUCCI reporters ubi-mRFP-NLS-CycB_1-266_ (red) and ubi-GFP-E2f1_1-230_ (green) together with DAPI (grey) in the areas outlined in yellow, and anti-DE-cadherin staining (blue). In *ttm2* expressing brains the lamina side of the NE (arrow) appears unaffected while the hyperplasic medulla side of the NE (arrowheads) presents green only cells in the most lateral side, and cells that express both the red and green tags at levels that are much higher than those found in wild-type NE in the most medial side. Scale bars, 50 µm in upper panels and 20 µm in insets.

To determine the cell cycle stage of the cells of the overgrown NE of *c855>ttm2* brain lobes we used Fly-FUCCI (fluorescent ubiquitination-based cell cycle indicator) [27]. The Drosophila FUCCI system relies on fluorochrome-tagged degrons from CycB (in red) and E2F1 (in green), which are degraded during mitosis and at the onset of the S phase, respectively. Consequently, Fly-FUCCI expressing cells are labelled green from anaphase to the G1-S transition, red in the S-phase, and yellow from G2 to early mitosis [27]. In wild-type lobes, most cells in the NE, both in the lamina and in the medulla sides (Figure 2A, arrow and arrowhead, respectively), present CycB-FUCCI (red) and E2F1-FUCCI (green), thus appearing yellow, which corresponds to G2 and early mitosis. Signal intensity is much stronger for both markers in the cells of the NE that are in the lamina side. Wild-type lamina and medulla cells are predominantly green (i.e., G1/S), except for some medulla cells near the NE that are predominantly red (i.e., S-phase) (Figure 2A).

In *c855>tm2* brain lobes, Fly-FUCCI staining in the lamina side of the NE remains mostly yellow, as in control brains (Figure 2B, arrow). However, ectopic Ttm2 has a conspicuous effect on the overgrown medulla side of the NE (Figure 2B, arrowhead) where two distinct regions can be identified along the lateral-to-medial axis. Most of the cells in the lateral side present red and green fluorescence at relatively low levels (i.e., pale yellow; G2), hence resembling those from wild-type NE, but some are distinctly green only (i.e., G1), which is seldom the case in wild-type brains. Cells in the medial side of *c855>tm2* NE present both the red and green fluorescent tags at levels that are much higher than those found in wild-type NE cells, and therefore appear strongly yellow (i.e., G2). Like in wild-type brains, lamina and medulla cells are predominantly green in *c855>tm2* brain lobes (i.e., G1), but the region of the medulla that is nearest to the NE and composed mostly of red only cells (i.e., S-phase) is much wider in *c855>tm2* that in control larvae (Figure 2B). Immunostaining with antibodies against Patj, Disc large (Dlg), Stardust (Std), and atypical Protein Kinase C (aPKC) showed that apico-basal polarity and columnar cell shape remain unchanged in the overgrown *c855>tm2* NE (Supplementary Figure S2).

These observations reveal that ectopic *ttm2* in the larval brain has a rather specific effect on the cells of the medulla side of the NE that overproliferate and fail to generate medulla NBs. As a result, the NE overgrows many cell diameters wider than normal. The resulting supernumerary NE cells maintain epithelial structure and most of them are in G2.

### 3.2. Wing Disc Cells Expressing either of the Testis Mitochondrial Proteins Ttm2 or Tomboy20 Induce Apoptosis and Invade Their Wild-Type Neighbours

To investigate the effect of Ttm2 and Tomboy20 in a different somatic epithelium we used *hh-Gal4* to express each of these proteins in the posterior compartment of the wing imaginal disc. We found that like *c855>ttm2* and *c855>tomboy20*, *hh>ttm2* and *hh> tomboy20* larvae present developmental delay and die as pupae.

**Figure 3:**
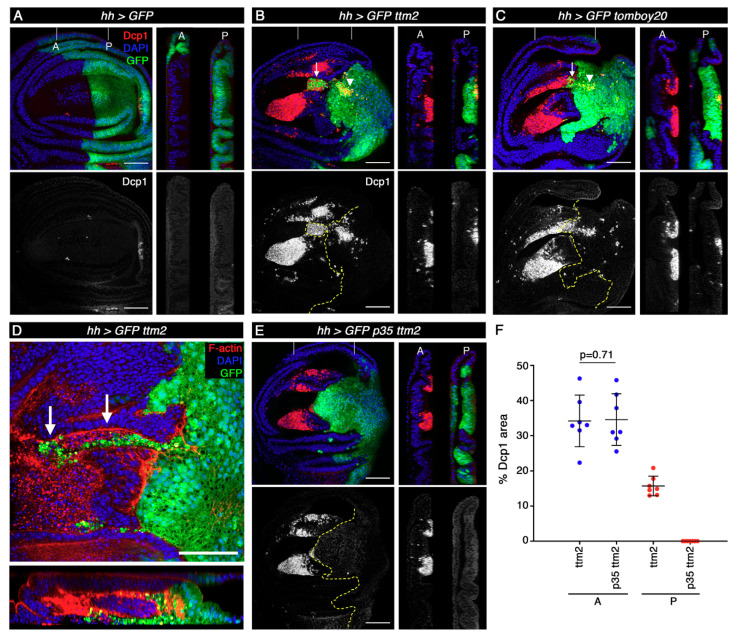
Ectopic expression of *ttm2* or *tomboy20* causes cell invasion and induces non-cell autonomous apoptosis. (**A**–**C**) XY and Z (anterior, A, and posterior, P) sections of control (*UAS-GFP/+; hh-Gal4/+* (*hh>GFP*)) wing disc (**A**), wing discs expressing *ttm2* (*UAS-GFP/+; hh-Gal4/UAS-ttm2* (*hh>GFP ttm2*)) (**B**), and wing discs expressing *tomboy20* (*UAS-GFP/+; hh-Gal4/UAS-tomboy20* (*hh>GFP tomboy20*) (**C**), stained with DAPI (blue) and anti-Dcp1 antibodies to reveal apoptotic cells (red in upper panels and grey in lower panels). In wild-type discs apoptotic cells are very rare. In *hh>GFP ttm2* and *hh>GFP tomboy20* discs, apoptosis levels in the posterior compartment are low (arrowheads), but there is a very high level of non-autonomous apoptosis in the anterior compartment. Moreover, most *hh>GFP ttm2* and *hh>GFP tomboy20* discs present posterior (green), strongly Dcp1 positive (red) cells that extrude basally and invade the anterior compartment (arrows). (**D**) XY and Z-sections of a wing disc expressing *ttm2* (*hh>GFP ttm2*) in the posterior compartment (GFP, green), stained with DAPI (blue) and Phalloidin (F-actin, red) showing a higher magnification view of the posterior cells that invade the anterior compartment (arrows). (**E**) Wing disc co-expressing *ttm2* and *p35* (*hh>GFP p35 ttm2*) in the posterior compartment (P, green), stained with DAPI (blue) and anti-Dcp1 antibodies (red, and grey). (**F**) Mean, SD, and scattered plot of the levels of apoptosis measured as the relative size of Dcp1 positive area in the anterior (A) and posterior (P) compartments of *hh>GFP ttm2*, and *hh>GFP p35 ttm2* wing discs (*n* = 7). Co-expression of *p35* eliminates all traces of cell autonomous apoptosis, but has no effect on the non-autonomous apoptosis that is induced in the anterior compartment. Vertical grey lines in XY panels show the position of the corresponding Z-sections. Dashed yellow lines show the A/P boundary. Scale bar, 50 µm.

We then carried out immunofluorescence with antibodies against the cleaved effector caspase Dcp-1 to determine the extent of apoptosis caused by the unscheduled expression of these proteins. As shown before, apoptosis levels are very low in the wild-type wing imaginal disc [28] (Figure 3A, red and grey). Both in *hh>ttm2* and *hh>tomboy20* wing discs, Dcp-1 levels are only moderately increased in posterior cells, and mostly focused on two patches: one is located in the posterior compartment, near the most distal part of the wing blade (Figure 3B,C; arrowheads); the other patch corresponds to a cluster of cells from the posterior compartment (GFP positive) that extrude basally and invade the anterior compartment (Figure 3B,C; arrows). Figure 3D shows a higher magnification view of these invasive cells.

In contrast to the moderate effect in the posterior compartment, Dcp-1 levels are massively increased in cells of the anterior compartment in *hh>ttm2* and *hh>tomboy20* wing discs, where the *hh-Gal4* driver is not active (GFP negative), even in areas that are located at a considerable distance from the anterior-posterior (A/P) boundary. As expected, Dcp-1 positive cells present distinctly heteropycnotic nuclei (Supplementary Figure S3). Most of these apoptotic cells concentrate on two large clusters located in the wing pouch that correspond to regions that are known to be more susceptible to apoptosis (Figure 3B,C; red and grey) [28,29]. These results demonstrate that unscheduled expression of *ttm2* or *tomboy20* in the wing disc epithelium brings about only a low level of cell-autonomous apoptosis, but causes a very high level of non-cell autonomous apoptosis in the wild-type neighboring cells.

These observations are reminiscent of the phenomenon known as “apoptosis-induced apoptosis” (AiA): apoptotic cells caused by the expression of *reaper (rpr)* or *head involution defective* (*hid)* in the posterior compartment release long-range death factors that induce apoptosis in the anterior compartment [30,31]. However, there are at least three notable differences between published AiA and our results. The first regards cell autonomous apoptosis that is massive upon expression of *rpr* or *hid* [30], but affects only a small fraction of the cells that express *ttm2* or *tomboy20*. The second difference is that, rather counterintuitively, the extent of induced apoptosis (i.e., non-cell autonomous apoptosis in the anterior compartment) is much greater in *hh>ttm2* and *hh>tomboy20* than what has been reported for *hh>rpr* and *hh>hid* discs [30]. A third and major difference between our observations and classical AiA is that co-expression of *ttm2* and *p35* from the *hh-Gal4* driver eliminates all traces of cell-autonomous apoptosis, but has no effect on the extent of induced apoptosis in the anterior compartment (Figure 3E,F). These results show that ectopic Ttm2 or Tomboy20 can, on its own, phenocopy the combined action of *p35* and *hid*/*rpr* expression (i.e., undead cells) as far as non-autonomous AiA is concerned. The same applies to cell invasion. *hid* expressing cells are not invasive, but undead cells delaminate basally and invade the wild-type neighbour tissue [31], thus resembling the patch of posterior cells that invade the anterior compartment in *hh>ttm2* and *hh>tomboy20* discs which, indeed, do not express *p35* (Figure 3B–D).

### 3.3. Ectopic Ttm2 or Tomboy20 Brings about Non-Cell Autonomous Overgrowth in the Wing Disc

**Figure 4:**
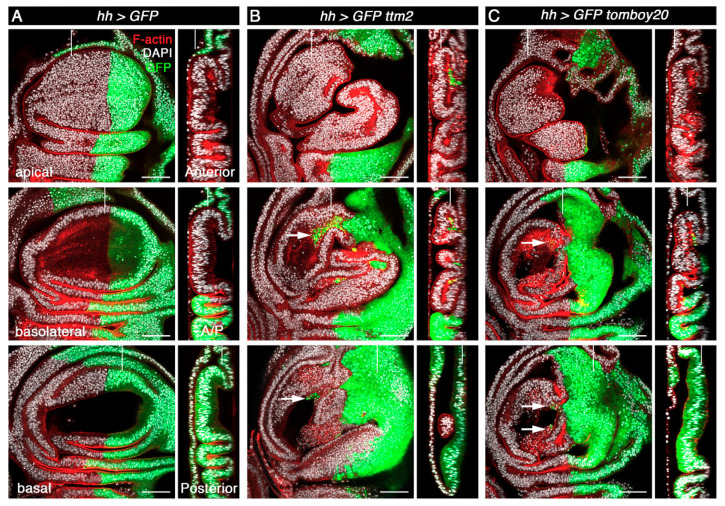
Ectopic expression of *ttm2* or *tomboy20* induces non-cell autonomous overgrowth. XY (apical, basolateral, and basal) and Z (Anterior, A/P and Posterior) sections of (**A**) control (*UAS-GFP/+; hh-Gal4/+* (*hh>GFP*)) wing disc, (**B**) wing discs expressing *ttm2* (*UAS-GFP/+; hh-Gal4/UAS-ttm2* (*hh>GFP ttm2*)) and (**C**) wing discs expressing *tomboy20* (*UAS-GFP/+; hh-Gal4/UAS-tomboy20* (*hh>GFP tomboy20*), stained with DAPI (grey) and Phalloidin (F-actin, red). The posterior compartment (green) in *hh>GFP ttm2* and *hh>GFP tomboy20* discs is flatter than the control (XZ sections, Posterior) and does not overgrow. In contrast, there is extensive non-autonomous overgrowth in the anterior compartment of the same discs, particularly in the hinge region. Cells from the posterior compartment that extrude basally and invade the anterior compartment are labelled with arrows. Vertical grey lines in XY and Z sections indicate the position of the Z and XY sections, respectively. Scale bar, 50 µm.

A distinct feature of co-expression of *rpr*/*hid* and *p35* from the *hh-Gal4* driver is both cell autonomous overgrowth in the posterior compartment as well as a low level of non-cell autonomous overgrowth in the anterior compartment that is limited to the region near the anterior/posterior (A/P) boundary [32,33,34,35,36]. In sharp contrast, we found that *hh>ttm2* and *hh>tomboy20* discs present overgrowth in the anterior compartment, notably in the wing base and hinge, but not in the posterior compartment (Figure 4B,C). Such a strong non-cell autonomous effect in overgrowth is yet a fourth difference between the effects caused by conditions that create undead cells and those caused by unscheduled expression of *ttm2* or *tomboy20* in the wing imaginal discs.

Expression of *ttm2* or *tomboy20* from the *hh-Gal4* driver also has a significant effect in the shape of the A/P boundary. Unlike control wing discs that present a smooth, straight boundary between the anterior and the posterior compartments (Figure 4A; GFP-negative and GFP-positive compartments) the A/P boundary in *hh>ttm2* and *hh>tomboy20* discs is highly convoluted (Figure 4B,C). Moreover, the posterior compartment wing blade epithelium is flatter in *hh>ttm2* and *hh>tomboy20* discs than in control *hh>GFP* discs and in the anterior compartment of *hh>ttm2* and *hh>tomboy20* discs (Figure 4B,C;Z posterior sections). None of these phenotypes are observed upon expression of *Tom20*, the somatic paralog of *tomboy20* from the *hh>Gal4* driver (*hh>Tom20*; Supplementary Figure S1). Basal delamination and flattened wing blade epithelium are also observed in discs that express *ttm2* all over the wing blade from the *nub-Gal4* driver (Supplementary Figure S5, *nub>ttm2*) that like the posterior compartment of *hh>ttm2* discs do not overgrow.

These results reveal that *ttm2* or *tomboy20* expressing cells do not overgrow themselves and induce massive non-autonomous overgrowth in the neighbor wild-type tissue.

### 3.4. Ectopic Ttm2 Expression in Wing Disc Cells Results in Enlarged Apical Surface and Nuclear Size, and Extended G2

**Figure 5:**
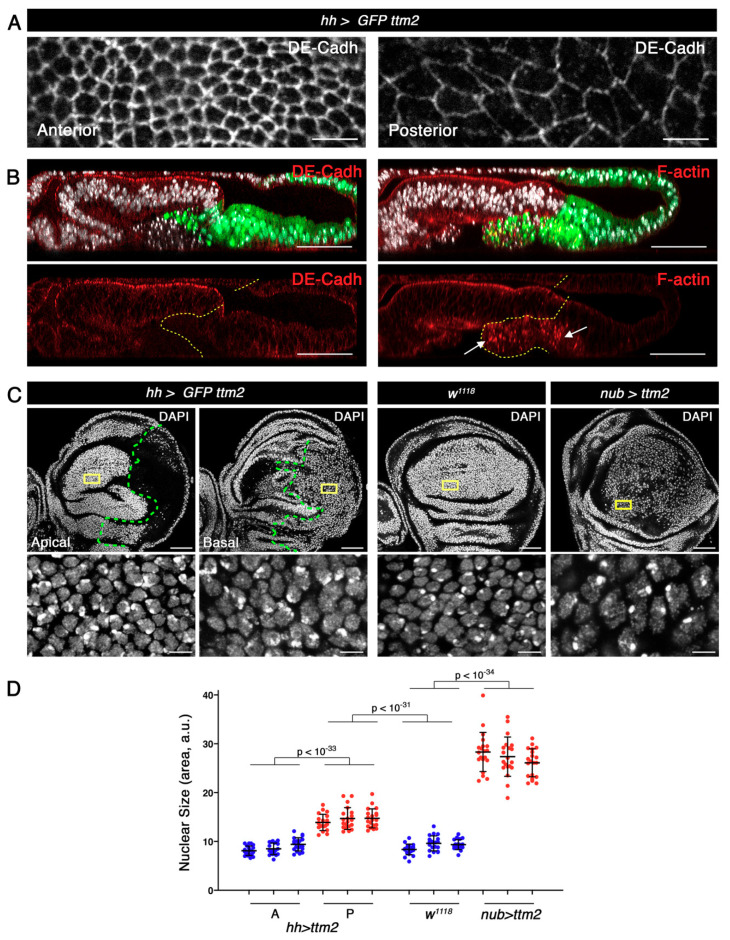
Ttm2 expressing epithelia present reduced levels of cell polarity and adhesion markers and enlarged cellular and nuclear sizes. (**A**) High magnification views of XY apical sections showing DE-Cadherin (DE-Cadh) staining (grey) in the anterior (left) and in the posterior (right) compartments in *UAS-GFP/+; hh-Gal4/UAS-ttm2* (*hh>GFP ttm2*) discs. In the posterior compartment the levels of DE-Cadh are notably reduced and cells present a greater apical surface. (**B**) Z sections show the posterior compartment (green), DNA (DAPI, grey), and DE-cadherin (left panels, red), or Phalloidin (F-actin, right panels, red). Dashed yellow lines label the A/P boundary. Discs are oriented with the peripodial membrane at the top and the columnar epithelium at the bottom. The levels of DE-Cadh and apical F-actin are notably reduced in the cells that express *ttm2* and remain in the epithelium. The cells that extrude from the posterior epithelium present a punctuated F-actin staining (arrows in **B**). (**C**) Wing discs expressing *ttm2* in the posterior compartment (*UAS-GFP/+; hh-Gal4/UAS-ttm2* (*hh>GFP ttm2*)), wild-type discs (*w^1118^*), and wing disc expressing *ttm2* in the entire wing blade (*nub-Gal4/+; UAS-ttm2*/+ (*nub>ttm2*)), stained with DAPI (grey). High magnification insets in the lower panels correspond to the regions outlined in yellow in the upper panels. Dashed green line label the A/P boundary. (**D**). Mean, SD, and scattered plot of nuclear sizes in cells from the anterior (A) and posterior (P) compartments of *hh>GFP ttm2*, control (*w^1118^*), and *nub>ttm2* discs. Ttm2-expressing cells present significantly larger nuclear sizes than control cells (*w^1118^* and anterior cells of *hh>GFP ttm2* discs). For each condition *n* = 60 nuclei (20 nuclei per disc; 3 discs). Scale bar, 50 µm in B and C upper panels, and 5 µm in A and C lowers panels).

Immunostaining of *hh>ttm2* wing discs with antibodies against DE-Cadherin (DE-Cadh), Patj, Sdt, aPKC, Crumbs (Crb), and Dlg revealed that all these polarity markers localise as expected, but signal intensity varies (Figure 5A,B and Supplementary Figure S5). While Patj and Sdt immunofluorescence appears unaffected, the levels of Crb and Dlg are slightly compromised and those of DE-Cadh and aPKC are notably reduced in the posterior compartment (Figure 5A,B and Supplementary Figure S5). Staining with fluorescent phalloidin showed that F-actin is also severely compromised in the posterior compartment: apical F-actin is greatly reduced in the cells that remain in the epithelium and presents a punctuated staining in the posterior cells that delaminate in *hh>ttm2* wing discs (Figure 5B). FasIII levels are also notably reduced (> six-fold) in *ttm2* expressing cells (62.8 ± 11.8, *n* = 30) compared to control cells of the anterior compartment (9.6 ± 1.9, *n* = 30, *p* < 10^−16^) (Supplementary Figure S5). These results suggest that cell adhesion may be compromised in the posterior epithelium.

Immunostaining with any of these markers together with DAPI staining also revealed that Ttm2 expressing cells in the posterior compartment present larger apical surface and nuclear size than control anterior cells (Figure 5A, and Supplementary Figure S5). Quantification of nuclear sizes is shown in Figure 5D. Mean nuclear size of *hh>ttm2* posterior cells (14.4 ± 1.9, *n* = 60) is significantly larger than that of controls cells both from the anterior compartment of the same discs (8.7 ± 1.3, *n* = 60; *p* < 10^−33^) and from wild-type discs (9.1 ± 1.3, *n* = 60; *p* < 10^−31^). The increase in nuclear size is even more dramatic in *nub>ttm2* cells (27.26 ± 3.7, *n* = 60, *p* < 10^−34^). Indeed, because “nuclear sizes” were measured as areas from nuclear sections, these figures underestimate actual nuclear volumes.

**Figure 6:**
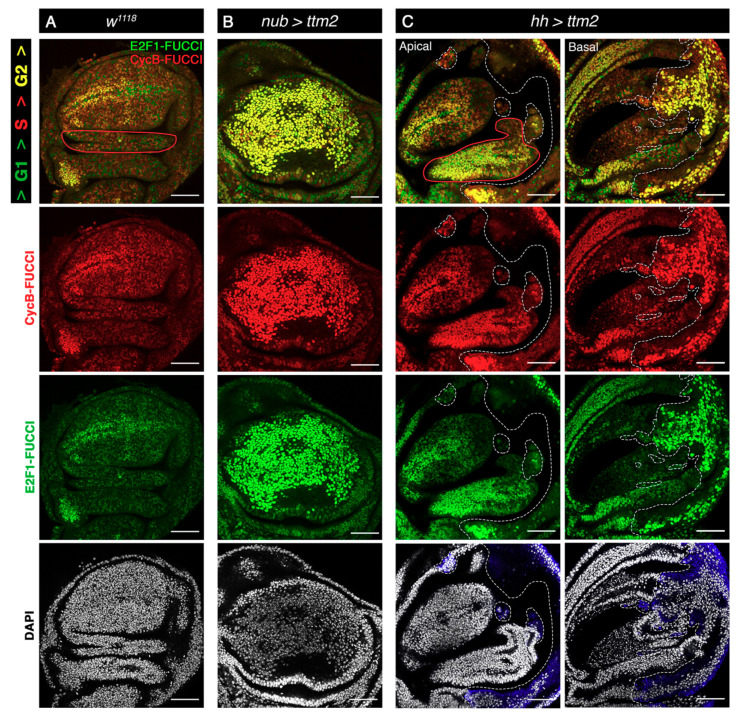
Ttm2 ectopic expression induces a significant extension of G2. (**A**) Control wing disc (*w^1118^*), (**B**) wing discs expressing *ttm2* in the entire wing blade (*nub-Gal4/+; UAS-ttm2*/+ (*nub>ttm2*)), and (**C**) wing discs expressing *ttm2* in the posterior compartment (*hh-Gal4/UAS-ttm2* (*hh>ttm2*)). All discs express the FlyFUCCI reporters ubi-GFP-E2f1_1-230_ (green) and ubi-mRFP-NLS-CycB_1-266_ (red) to label cells in G1 (green), G2/M (yellow), and S phase (red). DNA is labelled with DAPI (grey). The posterior compartment in *hh>ttm2* discs is labelled with antibodies against the HA tag of Ttm2 (blue). In *hh>ttm2* disc, apical and basal sections show the anterior and posterior compartment, respectively. The control disc (**A**) presents the normal cell cycle pattern. In contrast, *ttm2*-expressing cells in both *nub>ttm2* and *hh>ttm2* wing discs are intensely labelled in yellow, indicating a very significant G2 extension or arrest. A non-autonomous effect in cell cycle pattern is also observed in the hinge domain in the anterior compartment of *hh>ttm2* discs (outlined in red). Dashed white lines show the A/P boundary, as well as the boundaries between the patches of cells of posterior origin that have invaded the anterior compartment. Single confocal sections are shown. Scale bar, 50 µm.

We then determined the effect of Ttm2 ectopic expression in cell cycle progression using Fly-FUCCI. In wild-type discs Fly-FUCCI staining is most prominent in the zone of nonproliferating cells (ZNC) located in the dorso-ventral boundary (Figure 6A). The ZNC is composed of three subdomains (ventral, central, and dorsal), each about four-cells wide. Most cells in the anterior central subdomain and the entire posterior ZNC are in G1, while cells in the dorsal and ventral subdomains of the anterior ZNC are in G2 [37]. Cells in the wing pouch outside the ZNC and in the hinge proliferate in an unpatterned fashion and consequently, cells in these regions can be found at different cell cycles stages (Figure 6A).

Expression of Ttm2 exerts a severe effect in cell cycle patterns across the disc. In late third instar *nub>ttm2* discs, most cells appear strongly yellow because they contain high levels of both CycB-FUCCI (red) and E2F1-FUCCI (green) (Figure 6B). Mitoses are rare among these cells: the mitotic index determined as the relative frequency of phospho-Histone 3 (pH3) positive cells (0.51 ± 0.18, *n* = 10) is six to seven-fold lower (*p* = 0.0002) than that observed in control *nub>GFP* cells (3.32 ± 0.15; *n* = 6) (Supplementary Figure S6). The same applies to cells of the posterior compartment of *hh>ttm2* wing discs that present predominantly yellow Fly-FUCCI staining and a reduced mitotic index (0.69±0.29, *n* = 99) compared to that of control *hh>GFP* cells (5.27 ± 0.60; *n* = 7; *p* = 0.0002) (Figure 6C and Supplementary Figure S6). Altogether these results reveal that ectopic expression of *ttm2* in wing disc cells brings about a very significant extension of the G2 phase.

In addition to the described cell-autonomous effects, cell cycle progression is also conspicuously affected in the anterior compartment of *hh-Gal4>UAS-ttm2* discs where Fly-FUCCI yellow staining is prominent, particularly in the anterior side of the hinge, and the width of G2-stalled ventral and dorsal subdomains of the ZNC is enlarged (Figure 6C). Moreover, the intensity of both CycB-FUCCI and E2F1-FUCCI labels is severely reduced in the most posterior part of the anterior compartment of *hh>ttm2* discs. The mitotic index is three-fold lower in the anterior wing blade region of *hh>GFP* discs (1.34 ± 0.38, *n* = 9) than in the same region of control discs (3.68 ± 0.18, *n* = 7) (Supplementary Figure S6). These results reveal both autonomous and non-autonomous effects of Ttm2 expression in cell cycle progression in imaginal wing discs. They also show that an extended G2 and enlarged cell and nuclear sizes are separable events.

### 3.5. Ttm2 Expression Induces JNK Activation in Wing Discs, but Not in Neuroepithelia

**Figure 7:**
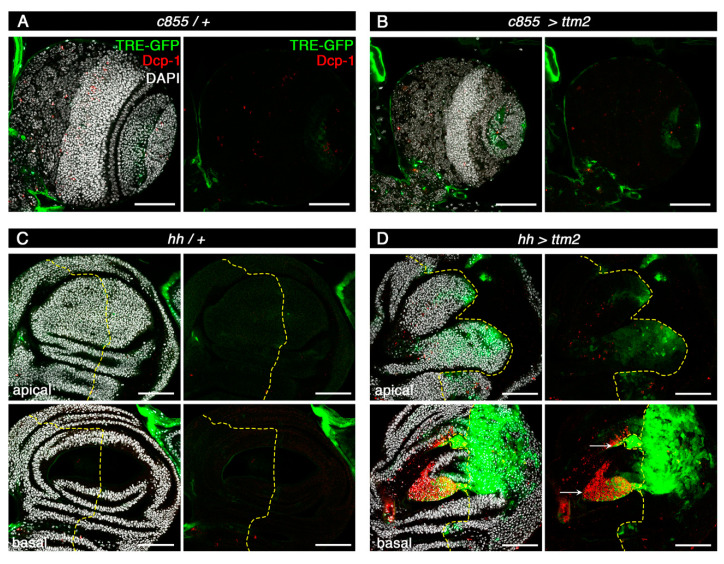
Ectopic Ttm2 induces JNK activity. (**A**,**B**) Control (**A**, *c855-Gal4/+* (*c855/+*)) and *ttm2*-expressing (**B**, *c855-Gal4/UAS-ttm2* (*c855>ttm2*)) larval brain lobes. (**C**,**D** Apical and basal sections of control (**C**, *hh-Gal4/+* (*hh/+*)) and *ttm2*-expressing (**D**, *hh-Gal4/UAS-ttm2* (*hh>ttm2*)) wing discs. Staining with DAPI, anti-Dcp1 antibody, and the JNK-reporter TRE-GFP are shown in grey, red, and green, respectively. TRE-GFP signal is at background levels in ttm2-expressing brains. In contrast, TRE-GFP signal is strong in *hh>ttm2* discs in the posterior compartment as well as near the A/P boundary (**D**, apical section), and in two large clusters of Dcp1 positive cells in the anterior compartment (**D**, basal section, arrows). Dashed yellow lines show the A/P boundary. Scale bar, 50 µm.

G2 stalling, enlarged cell size, and non-cell autonomous overproliferation in imaginal discs have been linked to JNK signaling [38], the activation of which is common to most of the published tumor models in this tissue (Reviewed in [39]). As a further step towards investigating the effect of ectopic *ttm2* we monitored the expression of the JNK signaling reporter TRE-GFP [40] in the NE and wing discs of *c855>ttm2* and *hh>ttm2* larvae, respectively (Figure 7). As expected, TRE-GFP fluorescence is not significantly above background levels in wild-type NE and wing discs. Interestingly, TRE-GFP expression levels remain equally low in the hyperplastic *c855>ttm2* NE. However, TRE-GFP is notably upregulated in *hh>ttm2* wing discs, both all over the posterior compartment, as well as in regions near the A/P boundary (Figure 7D, apical section) and in the two large clusters of Dcp1 positive cells (Figure 7D, basal section, arrows) in the anterior compartment. JNK signaling upregulates targets like Matrix metalloproteinase 1 (Mmp1) among others, that degrade the basement membrane and promote cell extrusion and invasion [41,42] which could readily explain the invasive behavior of *ttm2* expressing cells (Figure 3D).

These results strongly suggest that, as it does in many other tumor models, JNK could play a key role in *ttm2*-dependent non-autonomous apoptosis and overgrowth in wing disc epithelia [39]. However, while co-opting JNK signaling into promoting tissue growth can be readily explained in the presence of p35 or activated Ras85D that block apoptosis [43,44] it is not clear how the apoptosis promoting role of JNK is restrained in *hh>ttm2* wing discs. One possibility is that caspase activation in *ttm2*-expressing cells may be at threshold levels that are insufficient to direct fully penetrant cell death, but activate JNK and are optimal to induce cell migration [31]. Our results also strongly suggest that in contrast to wing discs, JNK activation may not be involved in *ttm2*-dependent cell-autonomous overgrowth in the NE.

## 4. Discussion

We do not know the mechanistic details of how ectopic *ttm2* brings about tumor growth in the NE and wing discs. Extended G2 and hyperplasia are common traits of the effect of ectopic upregulation of *ttm2* in these two epithelia. However, the mechanisms must be fundamentally different in these two epithelia because in wing discs, *ttm2* expressing cells bring about overproliferation in the neighboring cells (i.e., induced hyperplasia), while in the NE the *ttm2*-expressing cells themselves overproliferate (i.e., cell-autonomous hyperplasia). Moreover, ectopic *ttm2* triggers upregulation of JNK in imaginal discs, but not in the NE. These results demonstrate that a unique initial tumorigenic event may trigger different tumor growth pathways depending on the tissular context.

In the wing disc, the co-occurrence of G2-arrest, JNK activation, enlarged cell size, and non-cell autonomous overgrowth brought about by ectopic Ttm2 is closely reminiscent of the effect of *warts* (*wts*) and *discs large* (*dlg)* mutant clones that induce non-autonomous proliferation in the surrounding wild-type tissue [38]. Both mutant cells for either of these genes and neighbor wild-type cells upregulate TRE-GFP expression and present a strong G2 profile and increased cell size [38]. Interestingly, undead cells created by co-expressing *p35* and *egr* also arrest in G2 [38]. G2-stalling of JNK-signaling cells appears to be causative of the non-autonomous proliferation induced in the surrounding wild-type tissue by *wts* mutant cells because ectopic expression of *stg*, which is sufficient to suppress G2-stalling, significantly reduces wild-type tissue overgrowth [38]. These results were taken to suggest that transient cell cycle stalling in G2, which normally protects cells from JNK-induced apoptosis and is essential for wound healing, becomes detrimental upon chronic JNK overstimulation. Our results are fully consistent with this interpretation. Moreover, the hyperplastic growth brought about by ectopic Ttm2 in the NE identifies a different paradigm because G2 stalling is also prominent, but tissue overgrowth results from cell-autonomous overproliferation and JNK signaling does not appear to be involved.

Both *ttm2* and *tomboy20* have paralogs that are ubiquitously expressed: *ttm50* and *Tom20* [20]. This is not uncommon in Drosophila where a large fraction of gene duplicates present testis-enriched expression [45,46,47]. Such duplicates encode proteins that are involved in a wide range of functions. Examples of such duplicated genes are *twine* and *beta2-tubulin* that are expressed in primary spermatocytes, replacing their widely expressed homologues *cdc25/string* and *beta1-tubulin,* respectively [48,49,50,51]. There are also cases of such duplicates in mammals, but fewer than in flies [46,52,53]. As far as human CT genes are concerned, the only well-established case is “Brother of the Regulator of Imprinted Sites” (BORIS), which is the testis-specific paralog of epigenetic modulatory protein CCCTC-binding factor (CTCF). BORIS is normally expressed in testicular germ cells and repressed in somatic cells, but is aberrantly activated in different cancer types [54,55].

It has been reported that the effect of overexpression of *ttm50,* the ubiquitous paralog of *ttm2,* is increased apoptosis in the eye imaginal disc, and induced extra-proliferation, but only upon co-expression of *p35,* in wing discs [56]. Here, we show that overexpression of *Tom20,* the ubiquitous paralog of *tomboy20*, does not phenocopy Tomboy20 effects. Therefore, there is no evidence so far showing that the corresponding ubiquitous paralogues *ttm50* and *Tom20* may bring about hyperplasia when overexpressed in somatic tissues (where they are normally expressed). This, of course, does not rule out the possibility that either of them could be tumorigenic if expressed under different experimental conditions i.e., at a greater or lower rate, or in a different time window or tissue. Similarity and identity scores between Tomboy20 and Tom20 (58.5% and 36.8% over 171 residues, respectively) and between Ttm2 and Ttm50 (64.4% and 49.8% over 444 residues, respectively) are only moderate (Supplementary Figure S7). It has been proposed that a possible reason for such a divergence is that testis paralogs of somatic proteins evolve very rapidly [57]. Unfortunately, sequence differences do not suggest any hypothesis to explain the differential effect of ectopic expression of these germline genes and their ubiquitous paralogs in the wing disc. Indeed, the different tumorigenic effect of these two pairs of germline and ubiquitous paralogs cannot be taken to suggest in any way that germline-specific proteins in general may have a greater tumorigenic potential than ubiquitous (or somatic-specific) proteins.

Our results provide proof of principle and a new experimental model of the tumorigenic potential of ectopic expression of testis proteins in the soma.

## Supplementary Materials

The following are available online at https://www.mdpi.com/2073-4409/9/8/1842/s1, Figure S1: Ectopic expression of Ttm2 in larval brain lobes induces hyperplasia in the neuroepithelium. Figure S2: Apico-basal markers are not affected in *ttm2*-expressing neuroepithelia. Figure S3: Ectopic expression of *ttm2* produces non-cell autonomous apoptosis. Figure S4: Ectopic expression of *ttm2* produces cell delamination and a flattened wing blade epithelium. Figure S5: Localization and expression levels of apico-basal and cell adhesion markers in *ttm2*-expressing wing epithelium. Figure S6: The mitotic index is strongly reduced in *ttm2*-expressing discs. Figure S7: Sequence aligment of Ttm2 and Tomboy20 and the corresponding ubiquitous paralogs.
