# Supplementary material for: Context-Dependent Tumorigenic Effect of Testis-Specific Mitochondrial Protein Tiny Tim 2 in Drosophila Somatic Epithelia
Source: Cells. 2020 Aug 6;9(8):1842. doi: 10.3390/cells9081842 (PMC7465004; doi:10.3390/cells9081842)
Supplement: Supplementary file 1 [file cells-09-01842-s001.pdf]

cells

MDPI

Supplementary Figures

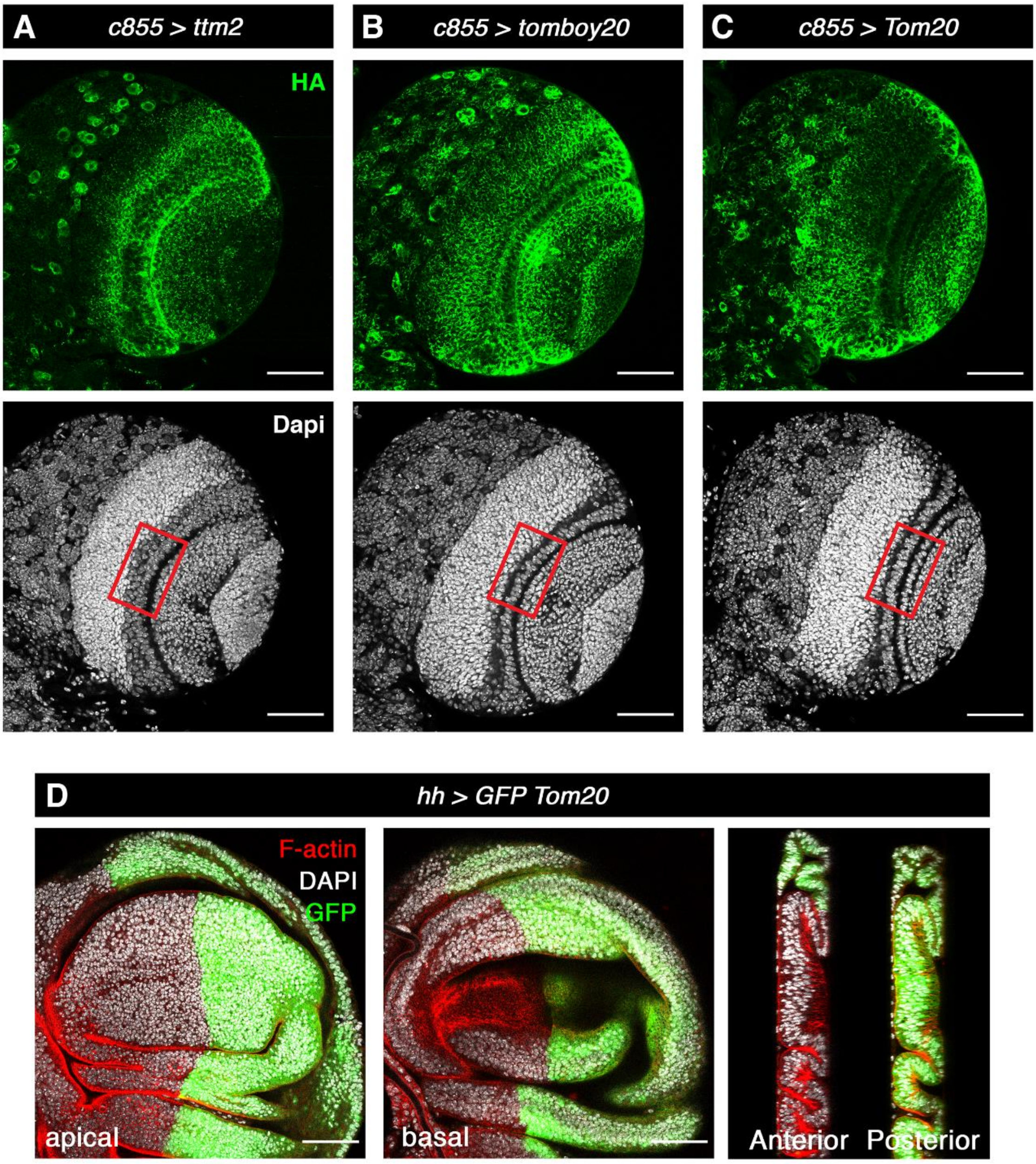

Supplementary Figure S1. Ectopic expression of Ttm2 in larval brain lobes, induces hyperplasia in the neuroepithelium. (**A–C**) Larval brain lobes expressing HA tagged *ttm2* (*c855>ttm2*) (**A**), *tomboy20* (*c855>tomboy20*) (**B**), and Tom20 *(c855>Tom20)* (**C**) stained with anti-HA antibodies (green) and DAPI (grey). HA tag signal (green) confirms the expression of the Ttm2, Tomboy20 and Tom20 proteins. The NE appears unaffected in *c855>tomboy20* or *c855>Tom20*, but is wider than normal in *c855>ttm2* brains. (**D**) XY (apical and basal) and Z (Anterior and Posterior) sections of a wing disc expressing *Tomboy20* ((*UAS-GFP/+; hh-Gal4/UAS-ttm2* (*hh>GFP ttm2*)), stained with DAPI (grey) and Phalloidin (F-actin, red). Scale bars, 50 µm.

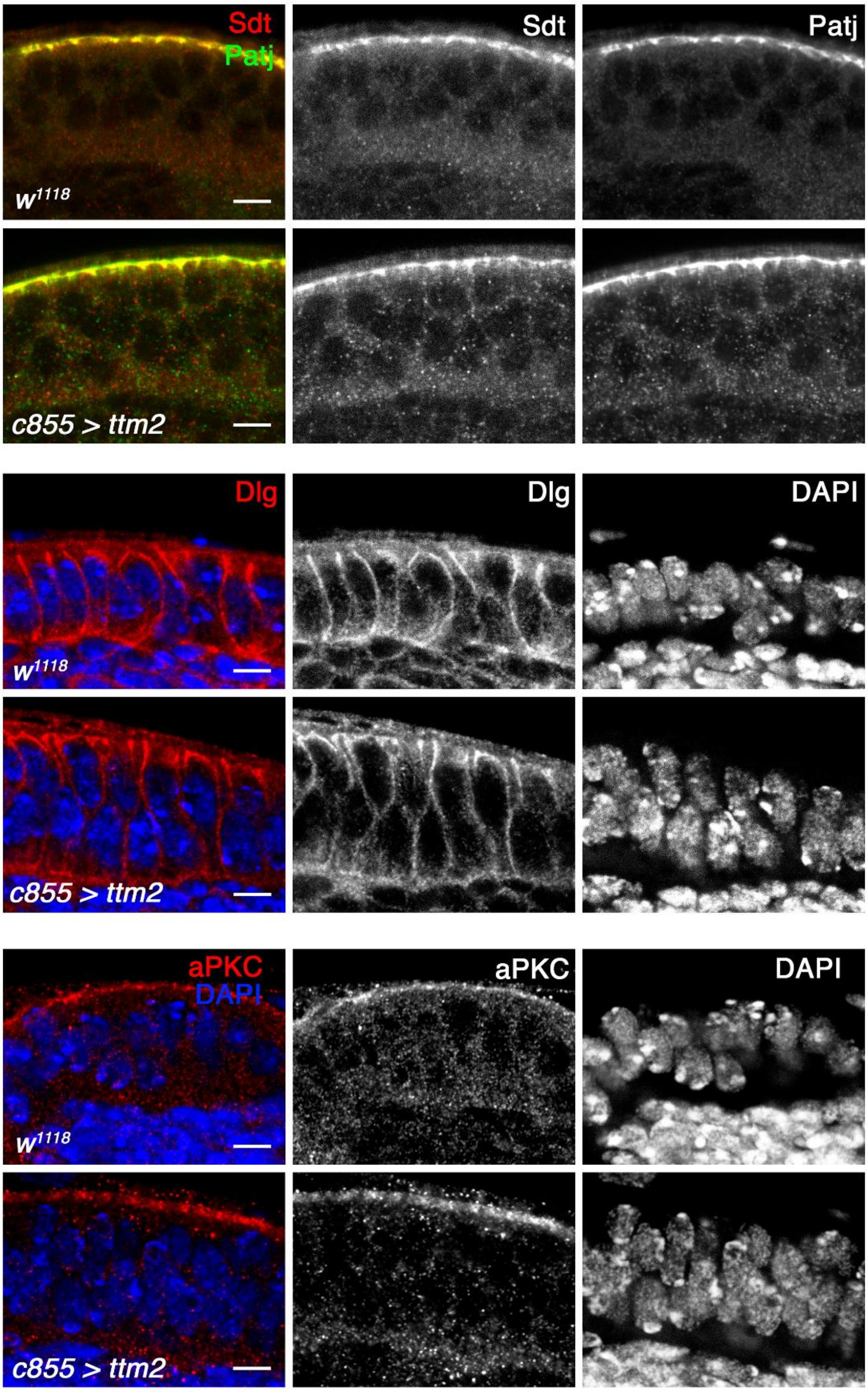

Supplementary Figure S2. Apico-basal markers are not affected in *ttm2*-expressing neuroepithelia. Cross sections of the NE of control (w^{1118}), and *ttm2* expressing brain lobes (*c855>ttm2*), stained with DAPI (blue) and antibodies against Patj, Sdt, Dlg, and aPKC. Cells in *c855>ttm2* NE maintain their columnar shape, and the normal levels and apical localization of Patj, Sdt, Dlg and aPKC. Scale bar, 5 µm.

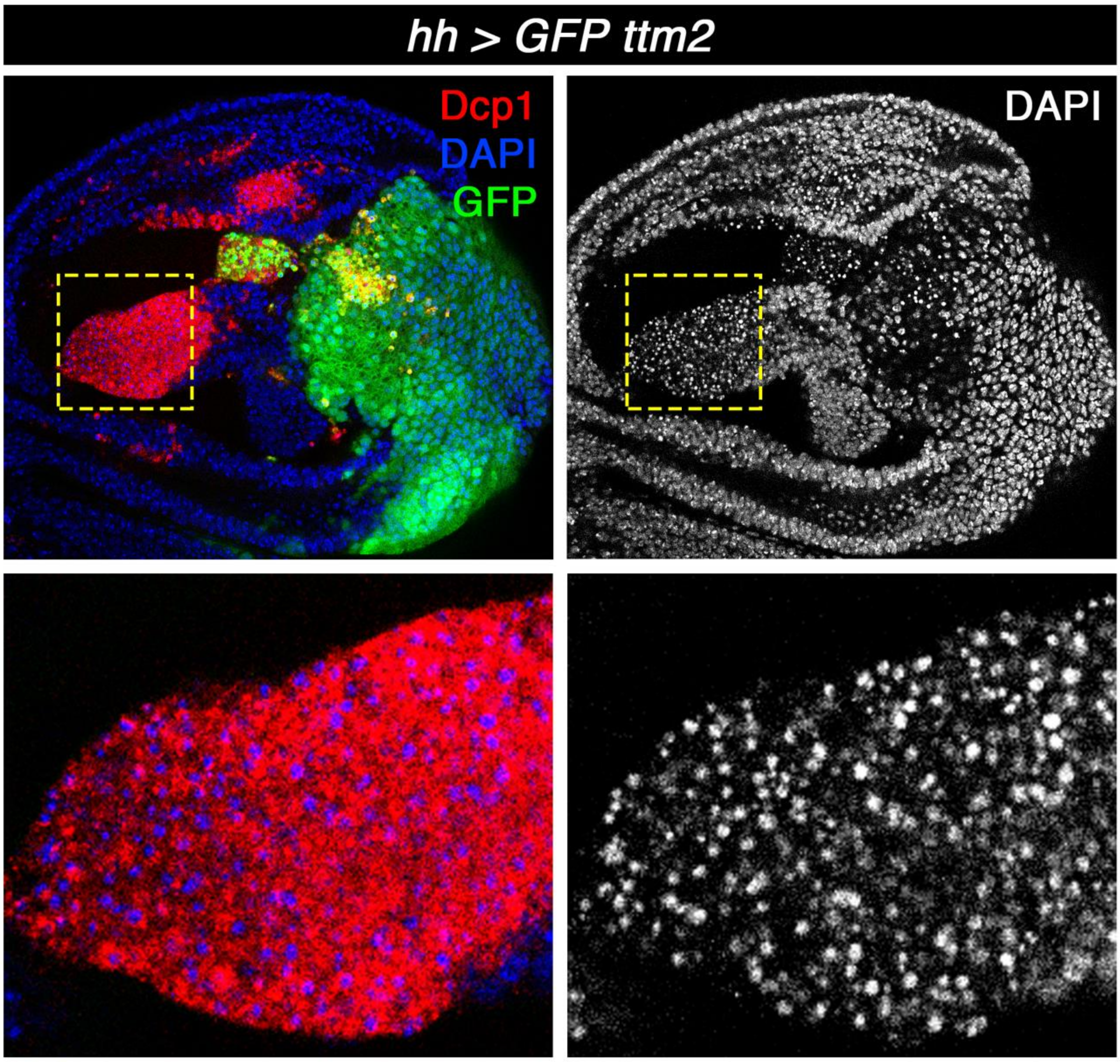

Supplementary Figure S3. Ectopic expression of *ttm2* produces non-cell autonomous apoptosis. Wing disc expressing *ttm2* in the posterior compartment (UAS-GFP/+; *hh-Gal4/UAS-ttm2* (*hh>GFP ttm2*)), stained with DAPI (blue and grey) and Dcp-1 (red). Most nuclei within Dcp-1 positive patches are highly heteropyknotic for DAPI. High magnification insets in the lower panels correspond to the region outlined in yellow in the upper panels.

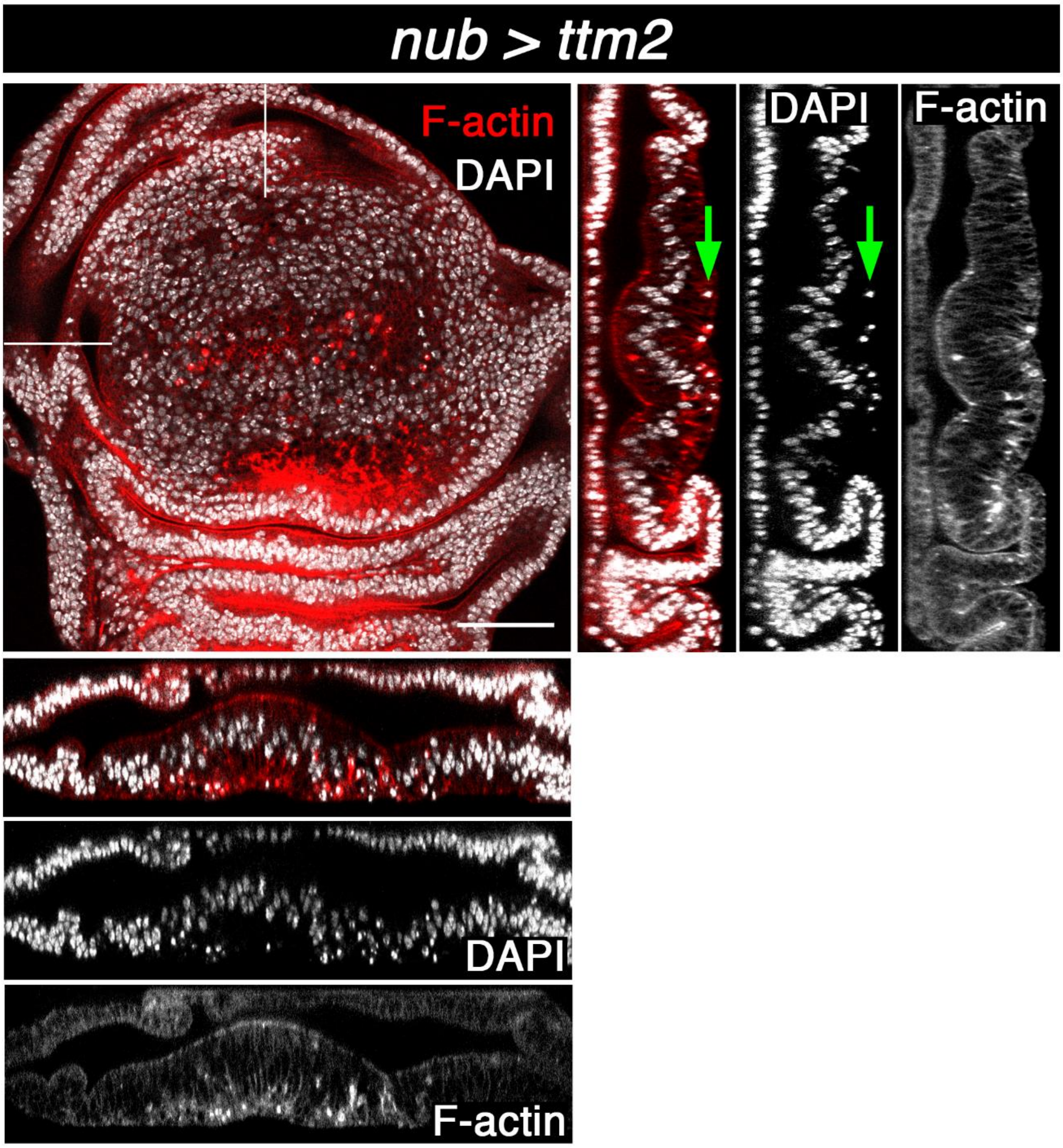

Supplementary Figure S4. Ectopic expression of *ttm2* produces cell delamination and a flattened wing blade epithelium. Wing disc expressing *ttm2* in the entire wing blade (*nub-Gal4*/+; *UAS-ttm2*/+ (*nub*>*ttm2*)), stained with DAPI (grey) and Phalloidin (F-actin, red). The white orthogonal lines in the XY section show the position of the corresponding Z sections. The *nub*>*ttm2* epithelium is flattener than normal. Cells that extrude basally are labelled (Z sections, green arrow).

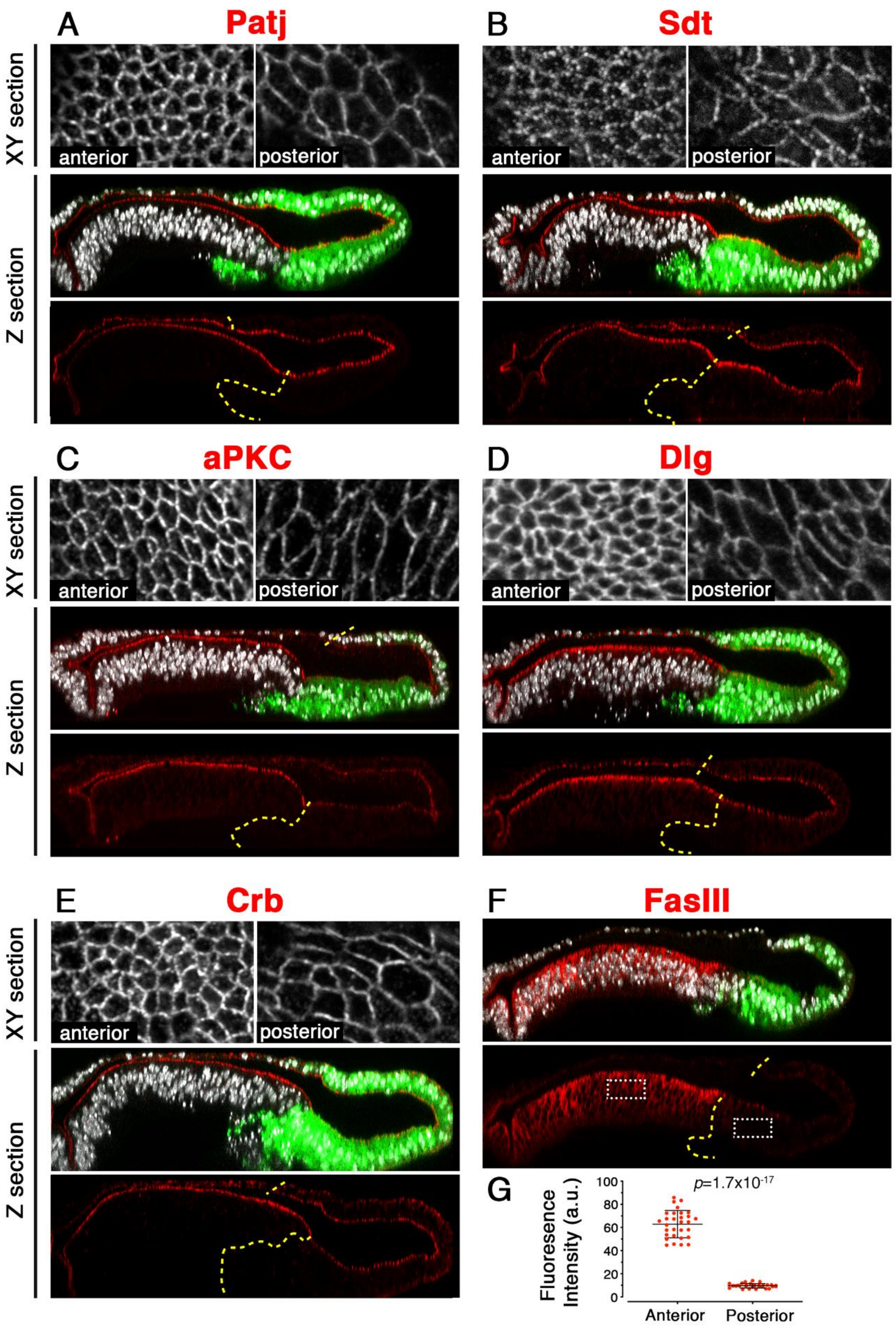

Supplementary Figure S5. Localization and expression levels of apico-basal and cell adhesion markers in *ttm2*-expressing wing epithelium. (**A–F**) XY apical sections, and XZ sections along the dorso-ventral boundary of wing discs expressing *ttm2* in the posterior compartment (*UAS-GFP/+; hh-Gal4/UAS-ttm2* (*hh>GFP ttm2*), stained with DAPI, and antibodies against Patj, Sdt, aPKC, Dlg, Crb, and FasIII. XY sections show high magnification views of the anterior and posterior regions stained

with the corresponding antibody (grey). XZ-sections show the staining with the corresponding antibody (red), DAPI (grey), and GFP (posterior compartment; green). Discs in these sections are oriented such that the peripodial membrane is at the top and the columnar epithelium at the bottom. Dashed yellow lines mark the A/P boundary. Patj and Sdt appear unaffected while the levels of aPKC, Dlg, Crb, and FasIII are reduced in ttm2 expressing cells. (**G**) Quantification of fluorescence intensity (measured in the outlined areas in F). Differences in FasIII levels between the anterior and the posterior compartment of *hh>GFP ttm2* wing discs (n=30) are highly significant. Mean with SD, and individual measurements are shown.

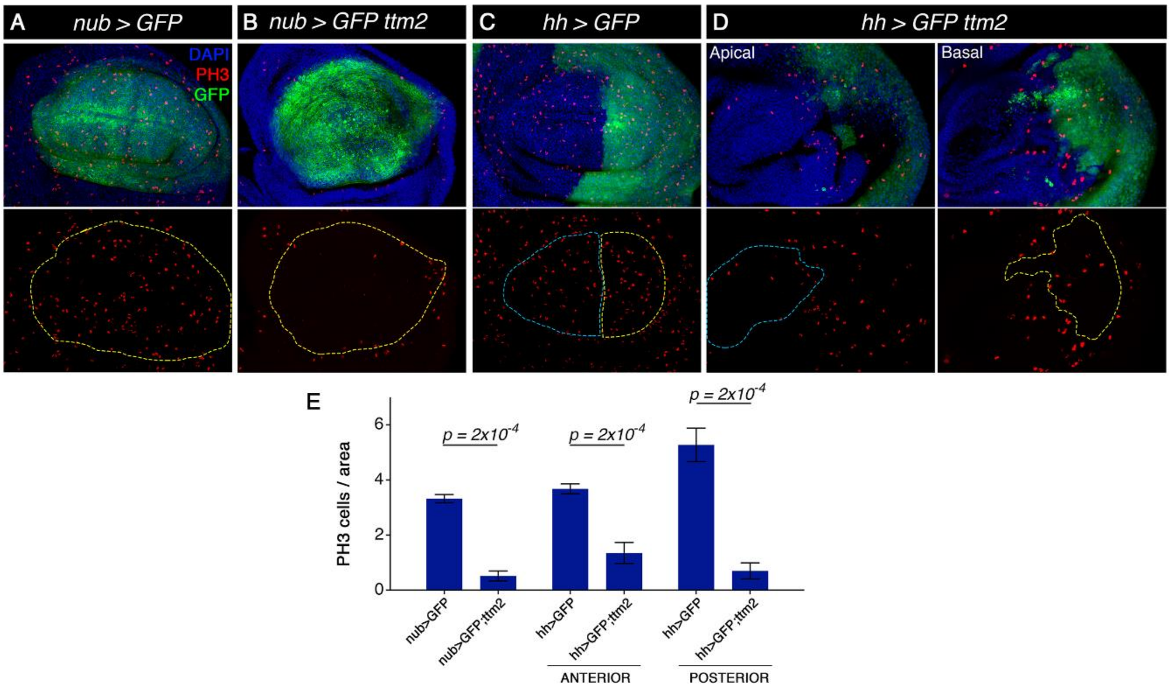

Supplementary Figure S6. The mitotic index is strongly reduced in *ttm2*-expressing discs. (**A**, **C**) Control wing disc (*nub>GFP* (*nub-Gal4/UAS-GFP*) and *hh>GFP* (UAS-GFP/+; *hh-Gal4*/+)), and (**B**, **D**) wing discs expressing *ttm2* (*nub>GFP ttm2* (*nub-Gal4/UAS-GFP; UAS-ttm2*/+) and *hh>GFP ttm2* (*UAS-GFP/+; hh-Gal4/UAS-ttm2*)) stained with DAPI (blue), and anti-PH3 antibodies (red). The area of *ttm2* expression is labelled by GFP fluorescence (green). (**E**) Mean and SD of the mitotic index in each of the conditions shown above, calculated as density of PH3-labelled cells in the outlined areas. The number of mitotic (PH3 positive) cells is significantly reduced in *nub>GFP ttm2* (n=10) compared to control *nub>GFP* discs (n=7). In *hh>GFP ttm2* discs (n=9) the number of cells in mitosis is significantly reduced in both anterior and posterior compartments compared to control anterior and posterior compartment of *hh>GFP* discs (n=7). Stacks of 10 focal planes per wing discs were obtained and the images were z-projected.

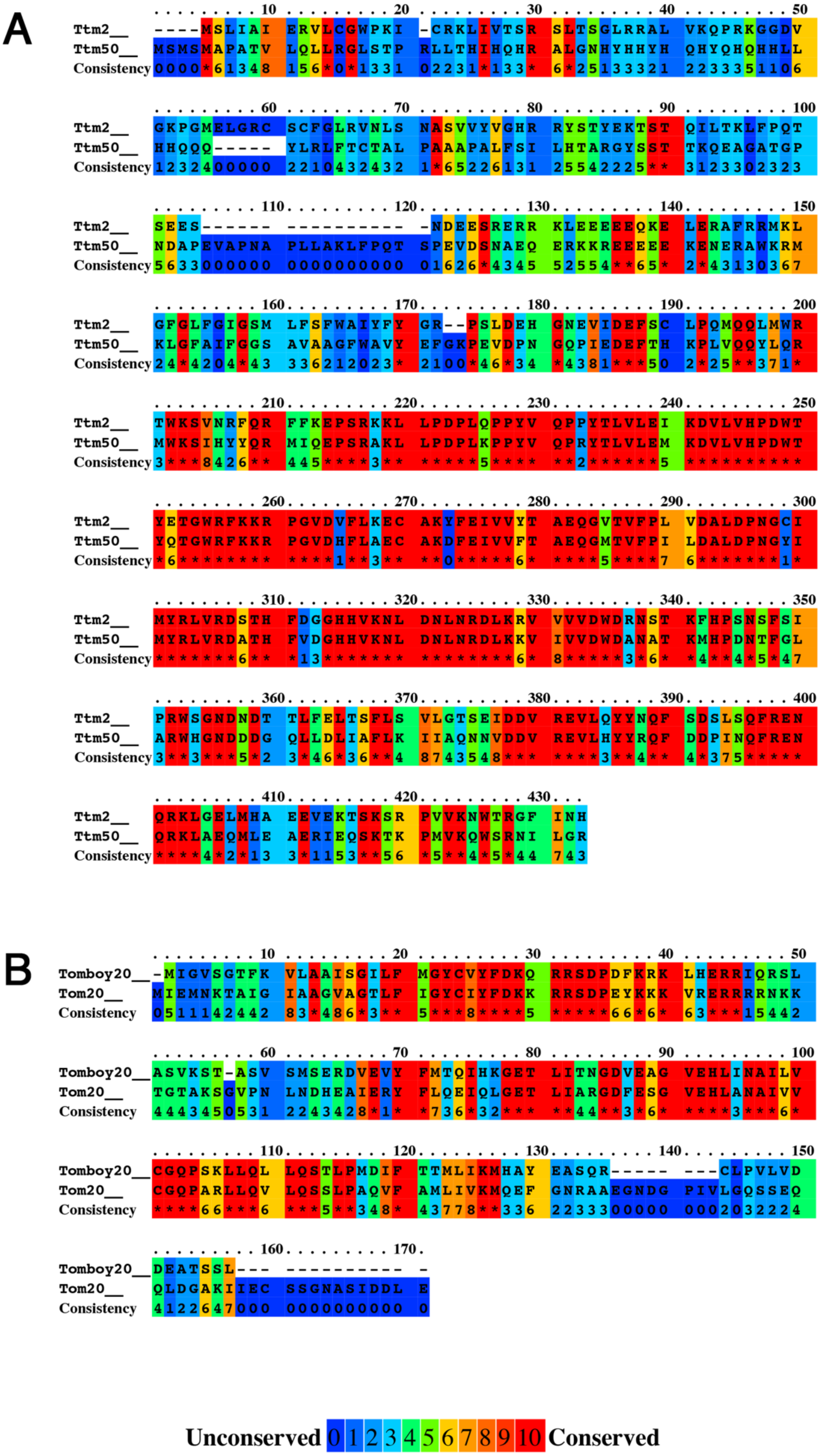

Supplementary Figure S7. Sequence aligment of Ttm2 and Tomboy20 and the corresponding ubiquitous paralogs. (**A**) **Ttm2 and Ttm50** and (**B**) Tomboy20 and Tom 20 protein alignment for amino acid conservation. The conservation scoring is performed by PRALINE software. The scoring scheme works from 0 for the least conserved alignment position, up to 10 for the most conserved alignment position.
